# Roles of UndA and MtrC of *Shewanella putrefaciens* W3-18-1 in iron reduction

**DOI:** 10.1186/1471-2180-13-267

**Published:** 2013-11-25

**Authors:** Yunfeng Yang, Jingrong Chen, Dongru Qiu, Jizhong Zhou

**Affiliations:** 1 State Key Joint Laboratory of Environment Simulation and Pollution Control, School of Environment, Tsinghua University, Beijing 100084, China; 2 Institute for Environmental Genomics and Department of Botany and Microbiology, University of Oklahoma, Norman, OK 73019, USA; 3 Institute of Hydrobiology, Chinese Academy of Sciences, Wuhan, Hubei 430072, China; 4 Earth Sciences Division, Lawrence Berkeley National Laboratory, Berkeley, CA 94720, USA

**Keywords:** *Shewanella putrefaciens* W3-18-1, Iron reduction, *c*-type cytochrome

## Abstract

**Background:**

The completion of genome sequencing in a number of *Shewanella* species, which are most renowned for their metal reduction capacity, offers a basis for comparative studies. Previous work in *Shewanella oneidensis* MR-1 has indicated that some genes within a cluster (*mtrBAC-omcA-mtrFED*) were involved in iron reduction. To explore new features of iron reduction pathways, we experimentally analyzed *Shewanella putrefaciens* W3-18-1 since its gene cluster is considerably different from that of MR-1 in that the gene cluster encodes only four ORFs.

**Results:**

Among the gene cluster, two genes (*mtrC* and *undA*) were shown to encode *c*-type cytochromes. The Δ*mtrC* deletion mutant revealed significant deficiencies in reducing metals of Fe_2_O_3_, α-FeO(OH), β-FeO(OH), ferric citrate, Mn(IV) and Co(III), but not organic compounds. In contrast, no deficiency of metal reduction was observed in the Δ*undA* deletion mutant. Nonetheless, *undA* deletion resulted in progressively slower iron reduction in the absence of *mtrC* and fitness loss under the iron-using condition, which was indicative of a functional role of UndA in iron reduction.

**Conclusions:**

These results provide physiological and biochemical evidences that UndA and MtrC of *Shewanella putrefaciens* W3-18-1 are involved in iron reduction.

## Background

A number of Gram-negative bacteria can grow anaerobically through dissimilatory reduction of metals such as insoluble Fe(III) and Mn(IV) oxides [1]. Among these, the genus of *Shewanella* has been a focus of research for its versatile capabilities of dissimilatory metal reduction, which has potentials for bioremediation of toxic metals [2-4]. Because of its metabolic capabilities, *Shewanella* is widely distributed in diverse habitats of soil, fresh water, marine water and even hydrothermal vents, with a preference of residing in stratified environments [2,5,6].

The most studied strain of *Shewanella* is undoubtedly *S. oneidensis* MR-1. It has been well established that some genes of an *mtrBAC-omcA-mtrFED* gene cluster of MR-1, such as *mtrBAC* and *omcA*, is involved in Fe(III), Mn(IV) and U (VI) reduction. This cluster contains two genes (*mtrC* and *omcA*) encoding outer membrane *c*-type cytochromes that form a protein complex [7] and function as a terminal reductase towards solid-phase metal (hydr)oxides. To facilitate the interaction with the solid-phase metal(hydr)oxides, these two cytochromes are organized in that MtrC is spatially distributed on cell surface while OmcA is localized between cell surface and minerals, as shown by antibody-recognition force microscopy [8]. Consistently, the presence of both MtrC and OmcA was required for reduction of solid-phase metal(hydr)oxides [9-11]. In comparison, not much is known about the specific functions of *mtrFED*. Recently, it was reported that Δ*mtrD* showed no deficiency in reducing soluble and insoluble Fe(III), but soluble Fe(III) reduction of the mutant was progressively slower when *mtrA* was also absent, implicating a role in Fe(III) reduction [12]. Similarly, Δ*mtrF* alone showed no deficiency in reducing soluble and insoluble Fe(III), but Δ*mtrF*/Δ*mtrC* was incapable of insoluble Fe(III) reduction.

The recent availability of whole genome sequences in dozens of *Shewanella* species has made it possible to examine the gene cluster of metal reduction in other members of the genus. Interestingly, the genome structure of the *mtrBAC-omcA-mtrFED* gene cluster is only conserved among closely related species of *S. oneidensis*[13]. To uncover variations in the molecular mechanism of iron reduction, here we report the characterization of this gene cluster in *S. putrefaciens* W3-18-1, which differs from *S. oneidensis* substantially in this gene cluster. In contrast to MR-1, which was isolated from the freshwater sediment of Lake Oneida, NY [14], W3-18-1 was isolated from a Pacific Ocean marine sediment off the coast of Washington State and originally characterized as a psychrophile that is able to reduce metals and form magnetite at 0°C [15]. We showed that MtrC (Sputw2623) was clearly involved in the reduction of Fe_2_O_3_, α-FeO(OH), β-FeO(OH) and ferric citrate, while deletion of a novel cytochrome gene (*undA* or *sputw2622*) resulted in progressively slower iron reduction in the absence of MtrC and fitness loss under the iron-using condition, indicating a role of UndA in iron reduction. Together, this work delineates a novel molecular mechanism of iron reduction in W3-18-1 that contrasts to what is known in MR-1.

## Methods

### Bacterial strains, plasmids, and culture conditions

A list of the bacterial strains and plasmids used in this study is described in Additional file 1: Table S1. *Shewanella* and *Escherichia coli* strains were grown aerobically in Luria-Bertani (LB) medium at 30 and 37°C, respectively [16,17]. When needed, antibiotics were added to growth media at the following final concentrations: Kanamycin (Kan), 50 μg/ml; ampicillin (Amp), 50 μg/ml; and gentamycin (Gm), 15 μg/ml. The suicide vector pDS3.0 has been described elsewhere [18]. Anaerobic medium was prepared by boiling the growth medium for 15 minutes with continuous purging with nitrogen gas. Then glass vials or bottles containing the medium were sealed with screw cap and butyl rubber septum followed by autoclave.

### Generation of in-frame deletion mutants

In-frame deletions of *mtrC, undA* or *mtrC-undA* genes in W3-18-1 were generated by the method of Link et al. [19]. In brief, PCR primers, as shown in Additional file 1: Table S2, were used to amplify 5′- and 3′- end fragments of *mtrC, undA* or *mtrC-undA* genes, respectively. The outside primers (D1 and D4) harbored a *Sac*I restriction site. The inside primers (D2 and D3) contained complementary 20-nt tags at their respective 5′ termini. Two fragments flanking *mtrC, undA* or *mtrC-undA* genes were amplified by PCR with corresponding primers D1 and D2, D3 and D4, respectively. Then PCR products were purified using the QIAquick PCR purification kit (Qiagen, Chatsworth, CA). Fusion PCR products were generated using the amplified fragments as templates with primers D1 and D4 as described elsewhere [19], then the fusion fragments were ligated into the *Sac*I site of plasmid pDS3.0 and the resulting mutagenesis plasmids (pDS-2622, pDS-2623, pDS-2622-2623, and pDS-4075) were transformed into the donor strain *E. coli* WM3064 [20]. The transformants were grown on LB supplemented with 0.3 mM diaminopimelic acid (DAP) and transferred to W3-18-1 by conjugation [21]. Integration of mutagenesis plasmids into the chromosome was selected by gentamycin resistance and confirmed by PCR amplification. Then transconjugants were grown in LB broth free of NaCl and plated on the LB plates supplemented with 10% of sucrose. Gentamycin-sensitive and sucrose-resistant colonies were screened by PCR to detect gene deletion, which was subsequently verified by DNA sequencing of the mutated region, and the deletion strain was designated as JZ2622(Δ*undA*), JZ2623(Δ*mtrC*) and JZ26223(Δ*mtrC-undA*).

### MtrC, UndA and MtrC-UndA complementation

For complementation, a 2.5-kb DNA fragment containing *mtrC* and its native promoter, a 2.9-kb DNA fragment containing *undA* and its native promoter, a 5.3-kb DNA fragment containing *mtrC* and *undA* and their native promoters were generated by PCR with W3-18-1 genomic DNA as the template (primers are listed in Additional file 1: Table S2). These fragments were digested with *Bam*HI and ligated to *Bam*HI-digested pBBR1MCS-2 to form pBBR1MCS-2-sputw2623, pBBR1MCS-2-sputw2622, and pBBR1MCS-2-sputw26223. Subsequently, plasmids were electroporated into WM3064 and introduced into the corresponding mutant by conjugation. Kanamycin-resistant colonies of the conjugants were selected for further examination. The presence of plasmids in the complementing strains was confirmed by plasmid purification and restriction enzyme digestion.

### Physiological and iron reduction measurement

Three replicates of strains were tested in all physiological experiments, which allows for two-way *t* test to determine the significance, and non-parametric dissimilarity test using adonis algorithm [22,23]. All physiological experiments were carried out under anaerobic condition with sodium lactate (20 mM, pH 7.0) as the electron donor, and ferric citrate (20 mM), α-FeO(OH) (20 mM), β-FeO(OH) (20 mM) or Fe_2_O_3_ (20 mM) as an electron acceptor. To set up the experiments, cultures were grown to exponential phase aerobically. Approximately ~10^5^ cells were transferred into anaerobic media above and kept still during anaerobic incubation.

The ferrozine assay was used to monitor Fe(III) reduction as previously described [24,25]. Iron reduction rates were calculated by dividing the differences of Fe(II) concentrations by the differences of time intervals.

### Heme stain

To detect the presence of *c*-type cytochromes, cells were grown anaerobically to the mid-log phase in LB medium supplemented with 50 mM sodium lactate, 20 mM fumarate and 10 mM ferric citrate and then centrifuged. The total cellular proteins were extracted from 0.2 ml cell culture using PeriPreps™ Periplasting kit (Epicentre, Madison, WI). The supernatant containing the cellular protein fraction was resuspended in SDS loading buffer and separated by SDS-PAGE using 12.5% polyacrylamide gels. Heme stains were performed using 3, 3′, 5, 5′-tetramethyl benzidine dihydrochloride as previously described [26].

### Competition assay

Competition assays were carried out to investigate the involvement of UndA in iron reduction. Wild-type, Δ*mtrC*, Δ*undA* and Δ*mtrC-undA* mutants were grown to exponential phase at OD_600_ of 0.6 aerobically. Equal volumes of culture were mixed together and inoculated by 1:100 dilutions into anaerobic LB medium supplemented with 50 mM sodium lactate and 20 mM ferric citrate. The co-cultures were transferred to fresh anaerobic medium in 1:100 dilutions on the daily basis. Samples were taken at Day one, three and seven and plated on LB plates aerobically. Colony PCR (96 colonies per plate, 3 replicates) with primers listed in Additional file 1: Table S2 was used to determine the ratios.

### Sequence analysis

Protein sequences were retrieved from the NCBI database by using BLASTP searches. The Clustal W software and the on-line tool Phylodendron (http://iubio.bio.indiana.edu/treeapp/treeprint-form.html) were used for the multiple alignment and phylogenetic tree construction.

## Results

### Comparison of iron reduction between *Shewanella putrefaciens* W3-18-1 and Shewanella oneidensis MR-1

W3-18-1 was shown previously to reduce Fe(III) oxide [27], which prompted us to conduct a comparison between W3-18-1 and MR-1 in reducing soluble or insoluble Fe(III) forms. To this end, the abilities of W3-18-1 and MR-1 in Fe(III) reduction were compared in liquid cultures supplemented with one of the following Fe(III) reagents as the sole electron acceptor: ferric citrate, α-FeO(OH), β-FeO(OH), and Fe_2_O_3_. All of the iron forms are insoluble except ferric citrate. α-FeO(OH), β-FeO(OH) and Fe_2_O_3_ are the major components of goethite, akaganeite and hematite, respectively.

**Figure 1:**
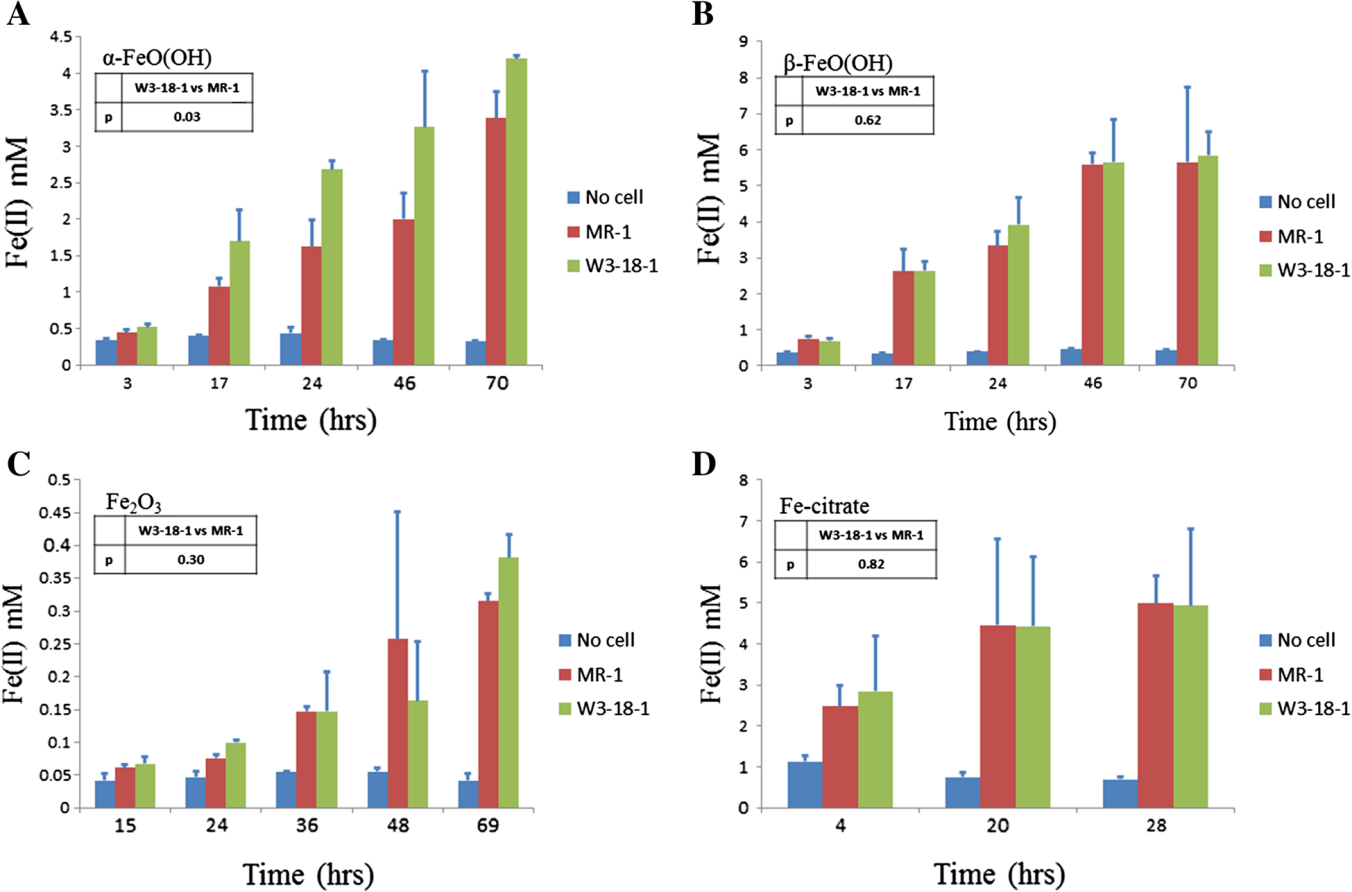
**Comparison of anaerobic (A) α- FeO(OH), (B) β- FeO(OH) (C) Fe**_**2**_**O**_**3 **_**and (D) ferric citrate reduction between MR-1 and W3-18-1.** A negative control was included, in which no bacterial cells were inoculated. Reduction of Fe(III) to Fe(II) was monitored using ferrozine at 562 nm. Data are averages for triplicates and error bars indicate standard deviation. The insets indicate significance of the dissimilarity test of adonis.

Across all of the five time points examined, W3-18-1 showed consistently higher iron reduction capacities than MR-1 when α-FeO(OH) was provided as electron acceptor (Figure 1). In contrast, iron reduction capacities with other iron forms were similar between W3-18-1 and MR-1. To verify it, a complementary non-parametric multivariate statistical test using adonis algorithm was carried out. The results indicated that the differences between W3-18-1 and MR-1 was significant for α-FeO(OH), but not other irons (see insets of Figure 1).

### Genes implicated in iron reduction

**Figure 2:**
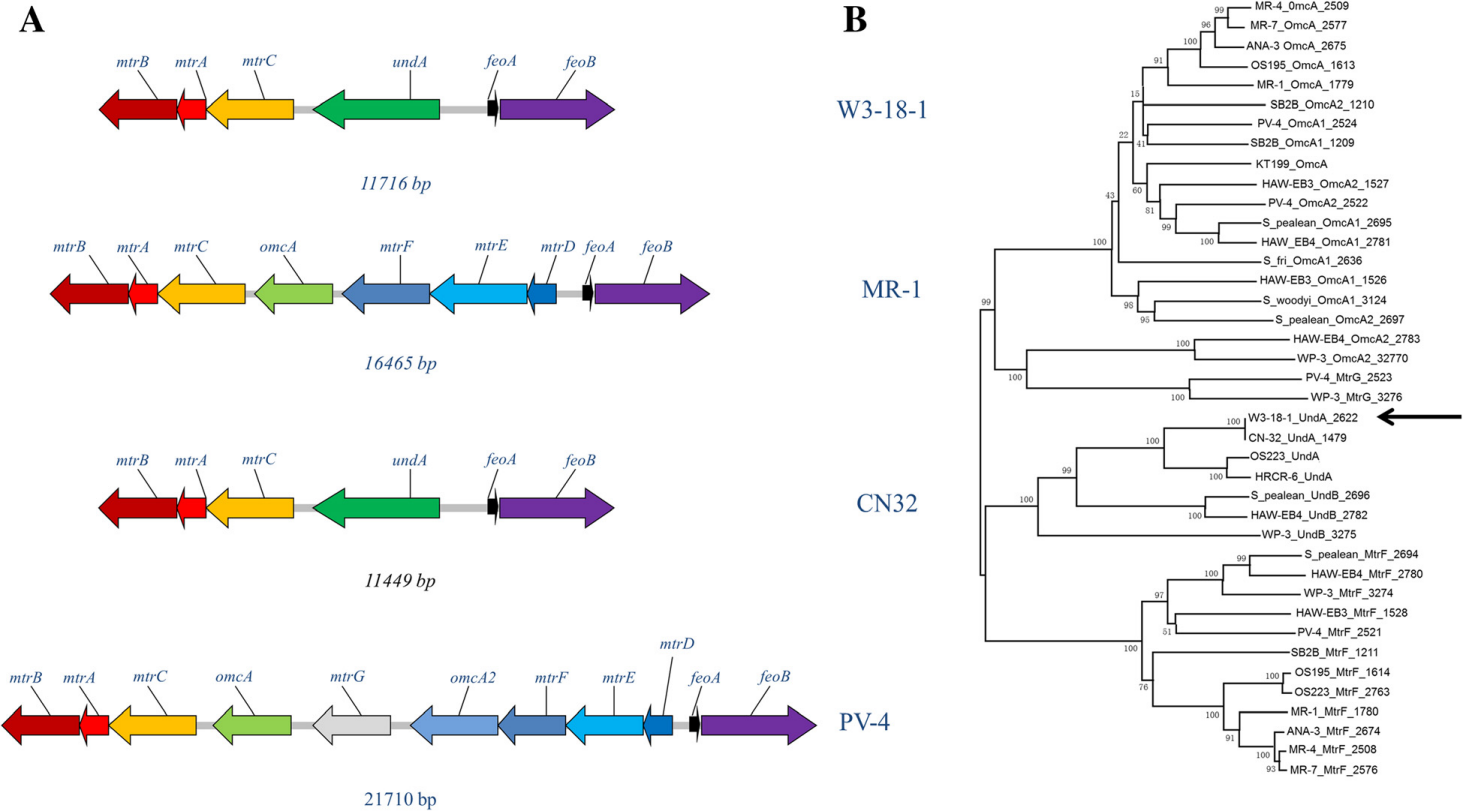
**Sequence analysis of *S.putrefaciens *W3-18-1 UndA. (A)** Schematic representation of the *mtr-omc* gene cluster in the genomes of selected *Shewanella* species. **(B)** The phylogenetic distance of UndA, MtrF, MtrG and MtrA protein sequences within sequenced *Shewanella*. The arrow points to the location of *S. putrefaciens* W3-18-1 UndA in the phylogenetic tree.

All of the currently sequenced *Shewanella* genomes except *Shewanella denitrificans* contain an *mtr-omc* gene cluster that encodes several proteins predicted to be associated with metal reduction [13,28]. Among these, *mtrBAC* are omnipresent and conserved in the cluster (Figure 2A). For example, a Blastp search indicated that W3-18-1 and *S. oneidensis* MR-1 MtrC share 48% identity and 60% similarity. However, W3-18-1 significantly differs from MR-1 in that the fourth gene of the gene cluster, designated as *undA* in this study, has no predictable orthologs in most *Shewanella* species. In addition, *S. oneidensis omcA* and *mtrDEF* are absent from the W3-18-1 genome. When protein sequence of *undA* was compared to that of *omcA* or *mtrF*, the results showed that it was 30% identity and 40% similarity, and 25% identity and 37% similarity, respectively. Notably, the identity between *undA* and *omcA* are largely attributed to the N-terminus (1–55 amino acids), which might be implicated as a signal peptide.

Conserved genomic synteny is noted for the *mtrBAC-undA* gene cluster. It is adjacent to a two-gene cluster comprised of *feoA* and *feoB*, which encode essential components of the Fe(II) transport system. The DNA interval between two gene clusters is 838 nucleotides.

To investigate the evolutionary aspect of UndA, the phylogenetic analysis of protein sequences was carried out. The results showed that UndA formed a small branch with its orthologs in *S. putrefaciens* CN32 and *S. baltica* OS223 (Figure 2B). It was also clustered with UndB, MtrF and MtrG, but separated from OmcA. Notably, the phylogenetic distance of UndA was substantially different from what has been reported from 16S rDNA sequences [29] or the whole genome [27].

### Phenotypes of W3-18-1 mutants

To characterize MtrC and UndA of W3-18-1, in-frame deletion mutants of Δ*mtrC* and Δ*undA* and a double mutant of Δ*mtrC-undA* were constructed. Furthermore, the ORF of *mtrC* or *undA* was tagged by six histidines, cloned onto an expression vector pBBR1MCS5 and transformed into the corresponding mutant, resulting in Δ*mtrC*- and Δ*undA*-complementing strains. The expression of MtrC and UndA in the complementing strains was verified by western blots using anti-his antibodies (data not shown).

A heme staining assay with mutant and complementing strains demonstrated that *mtrC* and *undA* encoded heme-containing proteins (Additional file 1: Figure S1). Genome annotation suggests that *mtrC* and *undA* encode a decaheme *c*-type cytochrome with a predicted molecular mass of 69 kDa and an eleven-heme *c*-type cytochrome with a predicted molecular mass of 88 kDa, respectively. Accordingly, there was no heme-positive band at a position corresponding to 88 kDa and 69 kDa in Δ*undA* and Δ*mtrC* mutant, respectively (Additional file 1: Figure S1A). Both bands were absent in the Δ*mtrC-undA* double mutant. Those missing bands in single mutants was restored in the complementing strains (Additional file 1: Figure S1B and S1C).

**Figure 3:**
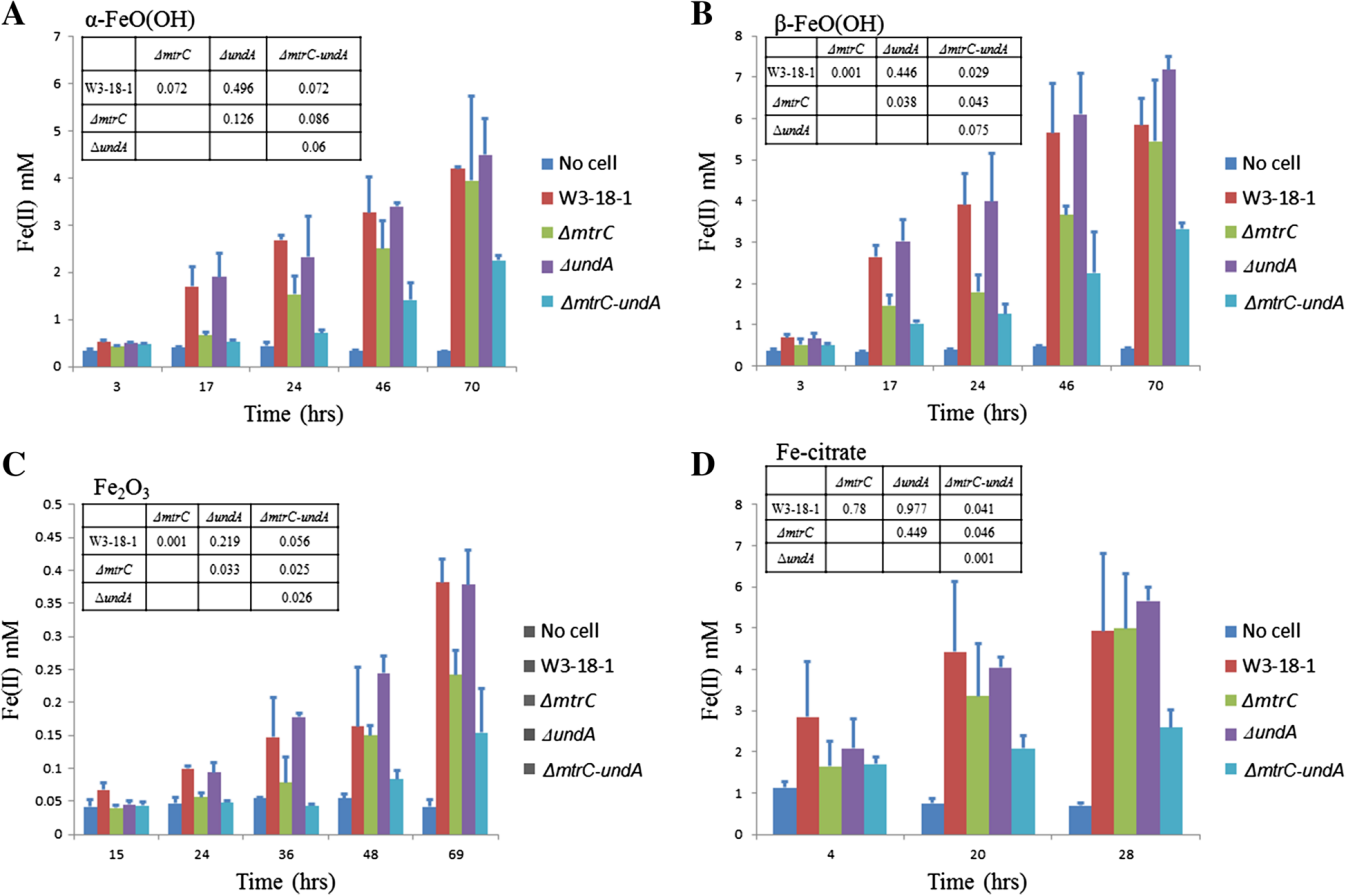
**Comparison of anaerobic (A) α- FeO(OH), (B) β- FeO(OH) (C) Fe**_**2**_**O**_**3 **_**and (D) ferric citrate reduction between W3-18-1 wild-type and Δ*mtrC*, Δ*undA *and Δ*mtrC-undA *mutants.** A negative control was included, in which no bacterial cells were inoculated. Reduction of Fe(III) to Fe(II) was monitored using ferrozine at 562 nm. Data are averages for triplicates and error bars indicate standard deviation. The insets indicate significance of the dissimilarity test of adonis.

The ability of ∆*mtrC* or ∆*undA* mutant to reduce Fe(III) was compared to that of the wild-type strain. When α-FeO(OH) was supplied, ∆*mtrC* mutant showed mild iron reduction deficiency (Figure 3A). In addition, significant (*P* = 0.001) deficiency was detected with β-FeO(OH) (Figure 3B) or Fe_2_O_3_ (Figure 3C) as the electron acceptor. When soluble ferric citrate was provided, no iron reduction deficiency was detected (Figure 3D). In contrast, similar iron reduction rates were detected for ∆*undA* mutant as compared to the wild-type strain (Figure 3), indicating that UndA was not required for iron reduction of W3-18-1.

Both ∆*mtrC* and ∆*undA* mutants were also examined for their ability of Mn(IV) reduction. Mn(IV), present as the insoluble form, could be reduced into soluble Mn(II) by W3-18-1. As shown in Additional file 1: Figure S2A, both wild-type and ∆*undA* mutant were similar in reducing insoluble Mn(IV) after 22 hour’s incubation, whereas the culture of ∆*mtrC* mutant remained turbid, which was indicative of Mn(IV) reduction deficiency. Furthermore, ∆*mtrC* mutant was also deficient in Co(III) (Additional file 1: Figure S2B). Therefore, ∆*mtrC* mutant was deficient in the reduction of multiple heavy metals. Together, these results suggested that *mtrC* deletion caused distinct deficiency of metal reduction in W3-18-1, whereas *undA* deletion had no detectable effects.

Also, we assessed the growth of ∆*mtrC* mutant under anaerobic conditions with 10 mM lactate as the electron donor, and one of the following four non-metal electron acceptors: 10 mM fumarate, 10 mM TMAO or 10 mM DMSO. The growth patterns were largely similar between wild-type and ∆*mtrC* mutant (Additional file 1: Figure S2C). Thus, in contrast to a role in metal reduction, MtrC appeared not to utilize organic compounds.

### The functional role of UndA in iron reduction

The ability of ∆*mtrC-undA* double mutant to reduce Fe(III) was examined. Iron reduction rates of ∆*mtrC-undA* double mutant appeared to be significantly lower than those of wild-type, ∆*mtrC* and ∆*undA* single mutants (Figure 3). The ∆*mtrC-undA* double mutant barely reduced any Fe(III) until 40 hours’ incubation when Fe_2_O_3_ was provided, whereas deficiencies were also notable when other Fe(III) forms were provided. Furthermore, iron reduction rates of ∆*mtrC-undA* double mutant were significantly lower than what was expected from the additive effects of MtrC and UndA single mutants (Additional file 1: Figure S3), indicating that UndA deletion resulted in slower iron reduction in the absence of MtrC.

**Figure 4:**
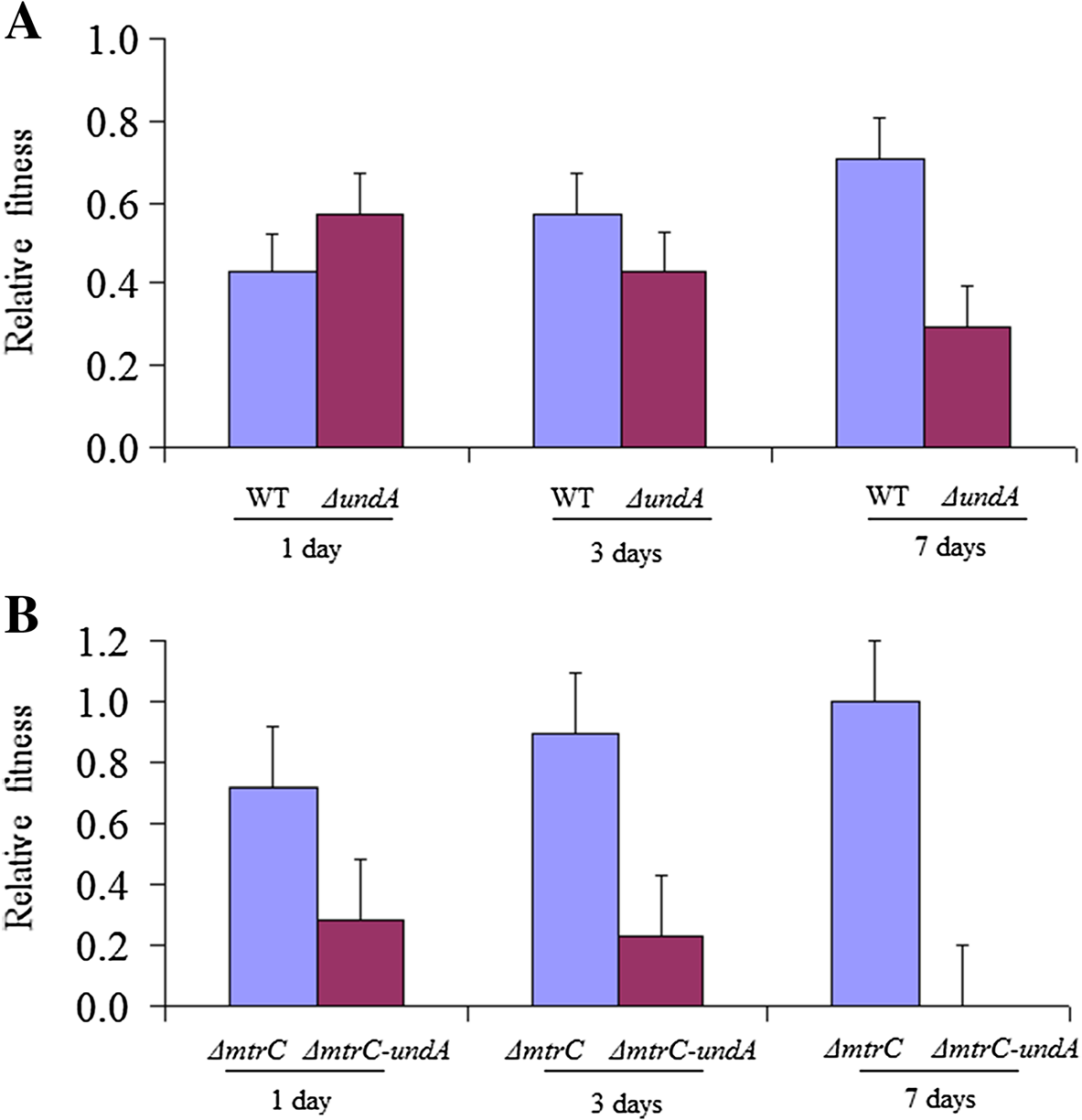
**The competition Assay for (A) wild-type (WT) vs. Δ*undA *and (B) Δ*mtrC *vs. Δ*mtrC-undA*.** Relative abundances of each strain in the co-culture at Day 1, 3 and 7 are shown.

To further demonstrate the functional role of UndA in iron reduction, competition assays were carried out to examine the fitness gain/loss caused by *undA* deletion. When wild-type and ∆*undA* cells were co-cultured in a medium with ferric citrate as the electron acceptor (Figure 4A), wild-type outcompeted ∆*undA* and gradually became dominant in the population by daily transfers. Similarly, Δ*mtrC* outcompeted Δ*mtrC-undA* (Figure 4B). These results indicated that UndA was needed to provide fitness advantage under iron-reducing conditions.

## Discussion

*Shewanella* are commonly present in redox stratified environments [13]. The successful establishment in such niches requires that bacteria adapt to utilize the electron donor or acceptor types in the environment. Accordingly, *Shewanella* strains are remarkable in utilizing a wide range of electron acceptors. Recent studies showed that *S. putrefaciens* W3-18-1 exhibited strong reduction of hydrous ferric oxide [30] as well as growth with DNA as sole carbon and energy source [31]. In addition, it could reduce metals and form magnetite at 0°C [15]. Here we further demonstrated that *S. putrefaciens* W3-18-1 was potent in reducing α-FeO(OH), ferric citrate, β-FeO(OH) and Fe_2_O_3_, which might be linked to the iron reduction gene cluster of W3-18-1. Notably, this gene cluster differs substantially from that of MR-1 in that it is comprised of only four genes (*mtrBAC* and *undA*) (Figure 2A). The mutational analysis in our study indicated that MtrC was specifically important for metal reduction (Figure 3 & Additional file 1: Figure S2), which was consistent with previous reports that its orthologs in other *Shewanella* strains played an important role in iron reduction [11,12]. In contrast, UndA was involved in, but not required for iron reduction. Based on these data, it appears that MtrC and UndA are primary and auxiliary components of iron reduction pathways, respectively.

Recent success in resolving the crystal structure of *Shewanella* sp. strain HRCR-6 UndA has revealed binding sites for soluble iron chelators [32]. Consistently, our iron reduction and competition experiments suggested that UndA was indeed involved in iron reduction. As a predicted outer membrane lipoprotein, *S. putrefaciens* UndA might directly interact with extracellular metals. A recent study showed that the UndA ortholog in *Shewanella* sp. strain HRCR-6 was secreted extracellularly by type II secretion system and participated in ferrihydrite and U(VI) reduction [33]. Interestingly, overexpressing UndA of HRCR-6 partially restored the iron reduction deficiency of Δ*mtrC-omcA* mutant. It is likely that overexpressing *S. putrefaciens* UndA could partially replace MtrC as well, since the promiscuity of outer membrane cytochromes can confer *Shewanella* an advantage to survive and thrive in the natural habitats [34]. Alternatively, *S. putrefaciens* UndA could function as an interchangeable module of MtrC in its interaction with other components in respiratory electron transfer reactions [12].

*S. putrefaciens undA* has no obvious orthologs in most *Shewanella* strains including *S. oneidensis* MR-1. Because comparative genomic analysis has revealed that UndA substitutes for OmcA in a number of *Shewanella* species [13,33], it is possible that UndA has a similar function as OmcA. However, our findings argued against this possibility, as mutant phenotypes of *S. oneidensis* OmcA differed substantially from those of W3-18-1 UndA in that *S. oneidensis* OmcA was important for Fe_2_O_3_ reduction and no linkage between OmcA and MtrC was detected under ferric citrate-reducing condition [12]. Rather, we noted that *S. oneidensis* Δ*mtrF* mutant displayed similar phenotypes as what were observed in our *S. putrefaciens* Δ*undA* mutant. It caused no deficiency of iron reduction, but progressively slower iron reduction in the absence of *S. oneidensis* MtrC [12]. These results suggested that *S. oneidensis* MtrF might function similarly as *S. putrefaciens* UndA. In support of this view, the overall structural fold of UndA is significantly similar to that of MtrF, despite low protein sequence identity [32,35].

## Conclusions

Comparative genomic studies have provided important clues into the gene diversity in the respiratory systems. Combining it with experimental studies brings us closer to understand the genetic variations of *Shewanella* genus. Using these approaches, we show in this study that UndA has a functional relatedness to MtrF, and MtrC and UndA play primary and auxiliary roles in iron reduction of W-3-18-1, respectively.

## Competing interests

The authors declare that they have no competing interests.

## Authors’ contributions

JC and DQ generated the constructs and strains used. YY, JC and DQ generated and analyzed the results. YY and JZ designed the study and drafted the manuscript. All authors read and approved the final manuscript.

## Supplementary Material

Additional file 1 Supplemental tables and figures associated with this manuscript. Click here for file
