# Supplementary material for: Roles of UndA and MtrC of Shewanella putrefaciens W3-18-1 in iron reduction
Source: BMC Microbiol. 2013 Nov 25;13:267. doi: 10.1186/1471-2180-13-267 (PMC4222724; doi:10.1186/1471-2180-13-267)
Supplement: Additional file 1 — Supplemental tables and figures associated with this manuscript. [file 1471-2180-13-267-S1.docx]

**Supplementary Information**

**Table S1**. Bacterial strains and plasmids used in this study

| Strain or plasmid | Description | Source or reference |
| --- | --- | --- |
| *E. coli* |  |  |
| WM3064 | Donor strain for conjugation; Δ*dap*A | ([Saltikov and Newman, 2003](#_ENREF_3)) |
| TOP10 | F2 *mcrA* Δ(*mrr-hsdRMS-mcrBC*) Φ80*lacZ*DM15 Δ *lacX74 deoR recA1 araD139* Δ(*ara-leu*)*7697 galU galK rpsL* (Sm^r^) *endA1 nupG* | Invitrogen |
|  |  |  |
| *Shewanella* |  |  |
| MR-1 | Wild-type strain | Lab stock |
| W3-18-1 | Pacific Ocean marine sediments isolate | ([Murray et al., 2001](#_ENREF_2)) |
| JZ2622 | *sputw2622* deletion mutant driven from W3-18-1; Δ*undA* (*sputw2622*) | This work |
| JZ2623 | *sputw2623* deletion mutant driven from W3-18-1; Δ*mtrC* (*sputw2623*) | This work |
| JZ26223 | *sputw2622* and *sptuw2623* double deletion mutant driven from W3-18-1; Δ*mtrC-undA* (*sputw2622-2623*) | This work |
| JZ2622P | *sputw4075* deletion mutant driven from Δ*undA* (*sputw2622*) | This work |
| JZ2623P | *sputw4075* deletion mutant driven from Δ*mtrC* (*sputw2623*) | This work |
| JZ26223P | *sputw4075* deletion mutant driven from Δ*mtrC-undA* (*sputw2622-2623*) | This work |
| JZ2622-COM | JZ2622P with pBBR1MCS-2-sputw2622 | This work |
| JZ2623-COM | JZ2623P with pBBR1MCS-2-sputw2623 | This work |
| JZ26223-COM | JZ26223P with pBBR1MCS-2-sputw2622-2623 | This work |
| JZ26223-BCOM | JZ26223P with pBBR1MCS-2-sputw2623 | This work |
| JZ26223-ECOM | JZ26223P with pBBR1MCS-2-sputw2622 | This work |
|  |  |  |
| Plasmids |  |  |
| PCR4-TOPO | 3.9-kb vector for cloning PCR products | Invitrogen |
| pDS3.0 | Suicide plasmid generated by cloning the gentamicin-resistance gene from pBSL142 into the *EcoR*V site of pCV442 | ([Yang et al., 2008](#_ENREF_4)) |
| pDS-2622 | pDS3.0 containing the PCR fragment for deleting *sputw2622* | This work |
| pDS-2623 | pDS3.0 containing the PCR fragment for deleting *sputw2623* | This work |
| pDS-2622-2623 | pDS3.0 containing the PCR fragment for deleting *sputw2622* and *sputw2623* | This work |
| pBAD/Thio | Amp^r^ vector for protein expression | Invitrogen |
| pBAD/Thio-2623 | pBAD/Thio containing *sputw2623* | This work |
| pBAD/Thio-2622 | pBAD/Thio containing *sputw2622* | This work |
| pBBR1MCS-2 | Km^r^ vector for complementation | ([Kovach et al., 1995](#_ENREF_1)) |
| pBBR1MCS-2-sputw2622 | pBBR1MCS-2 containing *sputw2622* from W3-18-1 | This work |
| pBBR1MCS-2-sputw2623 | pBBR1MCS-2 containing *sputw2623* from W3-18-1 | This work |
| pBBR1MCS-2-sputw26223 | pBBR1MCS-2 containing *sputw2622* and *sputw2623* from W3-18-1 | This work |

**Table S2**. Primers used in this study. Restriction digestion sites of in-frame deletion mutagenesis primers are underlined and the complementing 20-nt tags of primers are in bold.

| Primer Name | Sequence |
| --- | --- |
| *sputw2622* |  |
| D1 | TAGAGCTCgcgtgagtaaacagcagcag |
| D2 | **AATCATGTGACCCAGGCTTG**caacctgcgagtaggagtga |
| D3 | **CAAGCCTGGGTCACATGATT**aaacctgtgcaacctgtcaC |
| D4 | TAGAGCTCgcataggtttggcattgctt |
| Sf | cgacagagccttagcaatcc |
| Sr | tgccgttaaaatctccaagg |
| Lf | cgcccaaaaggttaatgaaa |
| Lr | ttttccctgcataggtttgg |
| *sputw2623* |  |
| D1 | GTTGCAGCTTGAGCTCCGT |
| D2 | **ATGAGCATGGAGGCATCTAT**TGCTAACAGCAGTGCGATTT |
| D3 | **ATAGATGCCTCCATGCTCAT**CAGGCAGTGCAGTCAGAAAC |
| D4 | ATTGGAGCTCGACAGGCTTC |
| Sf | TACACCCCTGGTAGCGAAAC |
| Sr | GCCAAAGAAATGGCAAGTGT |
| Lf | TAGCGGTCGTTTCGGTAACT |
| Lr | TTGGCTACGTGAACTTGGTG |
| *sputw2622-2623* |  |
| D1 | TAGAGCTCGCGTGAGTAAACAGCAGCAG |
| D2 | **TGGAATTGTGTCAGTGTCCC**AAAGTAAACCGGCACCCAAC |
| D3 | **GGGACACTGACACAATTCCA**AAAGGTGCATTGGAGATGAA |
| D4 | TAGAGCTCCCGCCCCTTTAGGAGAATAA |
| Sf | CGACAGAGCCTTAGCAATCC |
| Sr | CTCGGAGCAAGACACAATGA |
| Lf | CGCCCAAAAGGTTAATGAAA |
| Lr | ATTGGACCATGACAGGCTTC |

Mutant Complementation primers

| Primer Name | Sequence |
| --- | --- |
| Sputw2622-COM-F | CCGACGGATCCTTAGCAATC |
| Sputw2622-COM-R | CACCGAGGTGAGGGATCCTA |
| Sputw2623-COM-F | GGATCCTACACCCCTGGTAGCGAAAC |
| Sputw2623-COM-R | GGATCCGCCAAAGAAATGGCAAGTGT |
| Sputw26223-COM-F | CCGACGGATCCTTAGCAATC |
| Sputw26223-COM-R | GGATCCGCCAAAGAAATGGCAAGTGT |

Competition assay primers

|  | Forward | | Reverse |
| --- | --- | --- | --- |
| W3-18-1 vs. Δ*undA* | cgacagagccttagcaatcc |  | CACTCTGAAATTATCACTTG |
|  | CGACAGAGCCTTAGCAATCC | | tgccgttaaaatctccaagg |
| Δ*mtrC* vs. Δ*mtrC-undA* | TACACCCCTGGTAGCGAAAC | | GCCAAAGAAATGGCAAGTGT |
|  | CGACAGAGCCTTAGCAATCC | | CTCGGAGCAAGACACAATGA |

**Fig. S1**. Heme-stained SDS-PAGE profiles of total cellular protein extracted from the following strains: (A) W3-18-1 wild-type (WT), Δ*mtrC*, Δ*undA* and Δ*mtrC-undA*; (B) Δ*mtrC* and Δ*mtrC*-complementing strains; and (C) Δ*undA* and Δ*undA*-complementing strains. Bacterial cells were grown anaerobically to the mid-log phase in LB medium supplemented with 50 mM sodium lactate, 20 mM fumarate and 10 mM ferric citrate prior to total cellular protein extraction. The arrows indicate the migrating position of MtrC or UndA protein.

**
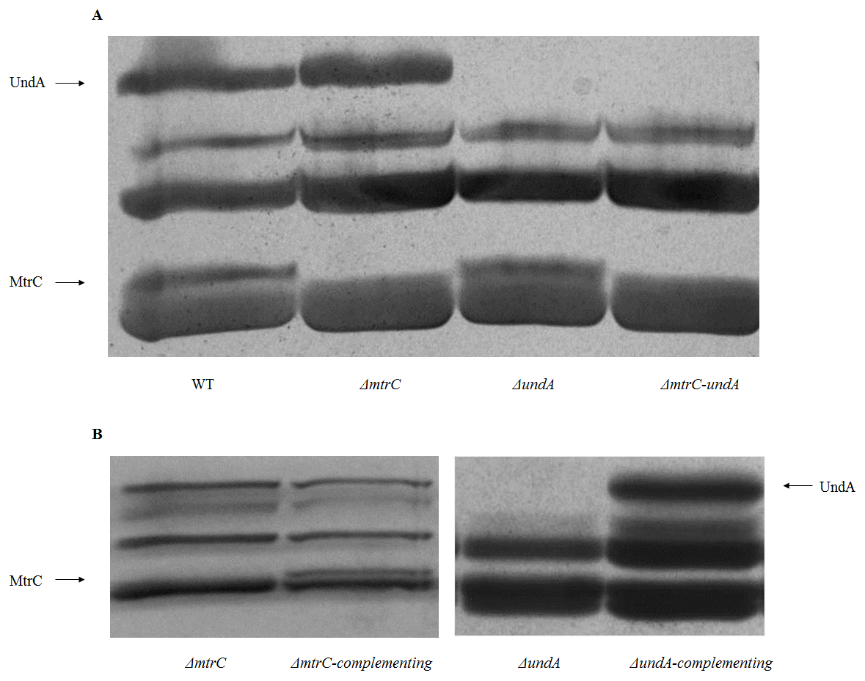
**

**Fig. S2.** (A) Reduction of Mn(IV) oxide by W3-18-1 wild-type (WT), Δ*undA*, Δ*mtrC* and Δ*mtrC-undA* mutants. (B) Reduction of Co(III) by WT and Δ*mtrC* mutant. (C) Anaerobic growth curves of WT and Δ*mtrC* mutant with fumarate, DMSO and TMAO as the electron acceptor. Data are averages for triplicates and error bars indicate standard deviation.


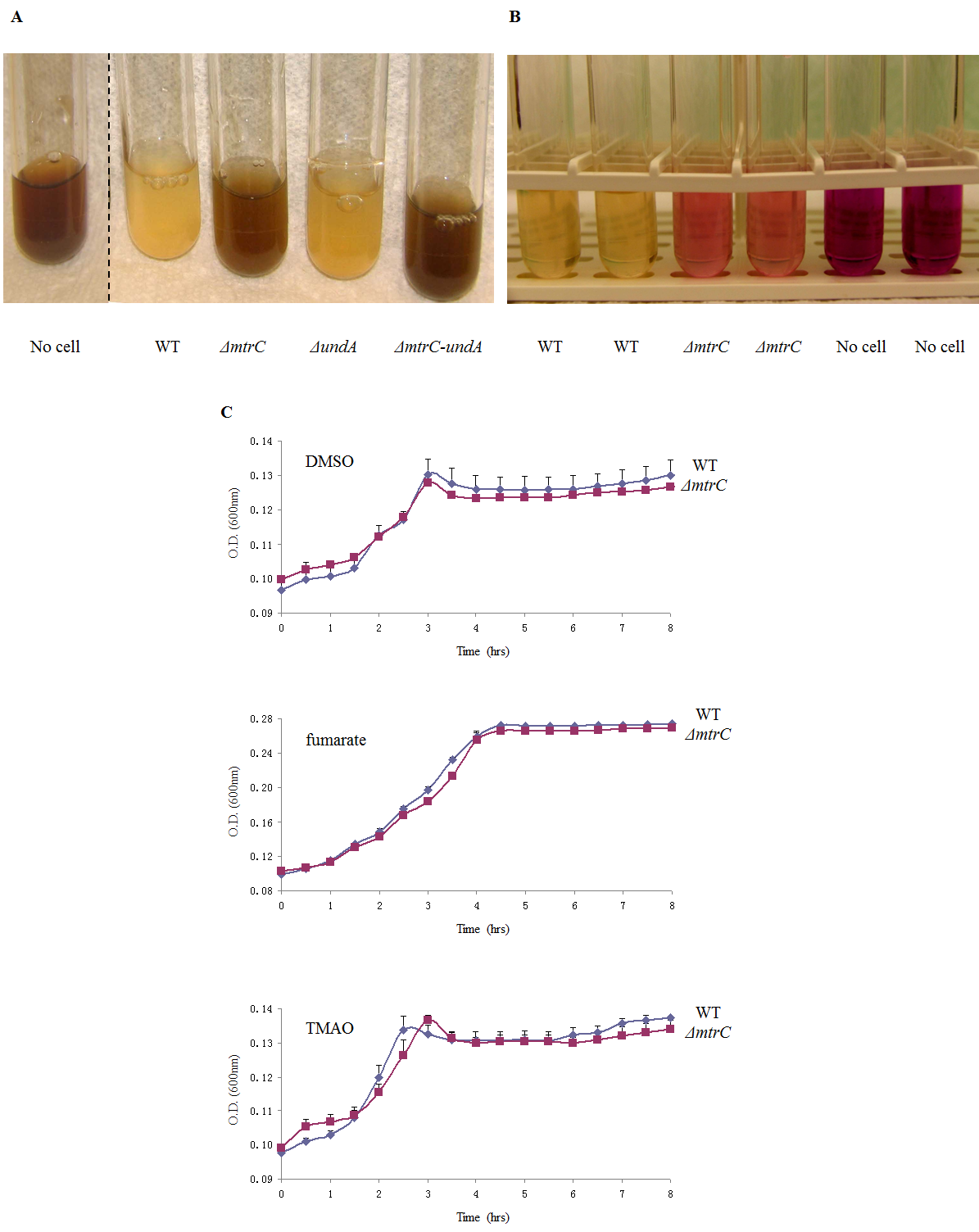


**Fig. S3.** Observed vs. additive effects of *mtrC* and *undA* deletion on iron reduction. The observed effect is the difference of iron reduction rates between Δ*mtrC-undA* and wild-type. The additive effect is the sum of the differences of iron reduction rates between Δ*mtrC* and wild-type, and Δ*undA* and wild-type.

**
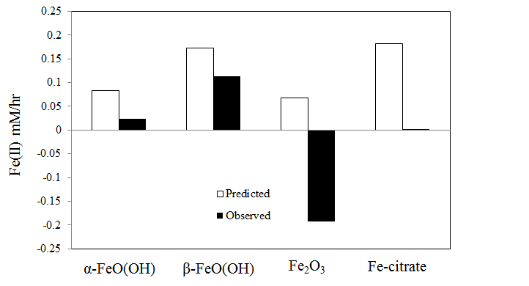
**

**References**

Kovach, M.E., Elzer, P.H., Hill, D.S., Robertson, G.T., Farris, M.A., Roop, R.M., 2nd, Peterson, K.M., 1995. Four new derivatives of the broad-host-range cloning vector pBBR1MCS, carrying different antibiotic-resistance cassettes. *Gene* **166**, 175-176.

Murray, A.E., Lies, D., Li, G., Nealson, K., Zhou, J., Tiedje, J.M., 2001. DNA/DNA hybridization to microarrays reveals gene-specific differences between closely related microbial genomes. *Proc Natl Acad Sci U S A* **98**, 9853-9858.

Saltikov, C.W., Newman, D.K., 2003. Genetic identification of a respiratory arsenate reductase. *Proc Natl Acad Sci U S A* **100**, 10983-10988.

Yang, Y., Harris, D.P., Luo, F., Wu, L., Parsons, A.B., Palumbo, A.V., Zhou, J., 2008. Characterization of the *Shewanella oneidensis* *fur* gene: roles in iron and acid tolerance response. *BMC genomics* **9** Suppl 1, S11.
